# In Silico CRISPR-Cas-Mediated Base Editing Strategies for Early-Onset, Severe Cone–Rod Retinal Degeneration in Three *Crumbs homolog 1* Patients, including the Novel Variant c.2833G>A

**DOI:** 10.3390/genes15050625

**Published:** 2024-05-15

**Authors:** Hoda Shamsnajafabadi, Maria Kaukonen, Julia-Sophia Bellingrath, Robert E. MacLaren, Jasmina Cehajic-Kapetanovic

**Affiliations:** 1 Nuffield Laboratory of Ophthalmology, Department of Clinical Neurosciences, Oxford University, Oxford OX3 9DU, UK; hoda.shamsnajafabadi@ndcn.ox.ac.uk (H.S.);; 2 Oxford Eye Hospital, Oxford University NHS Foundation Trust, John Radcliffe Hospital, Oxford OX3 9DU, UK

**Keywords:** *Crumbs homolog 1* (*CRB1*), CRISPR-Cas mediated base editing, cone–rod retinal degeneration, Müller glial cell degeneration

## Abstract

Pathogenic variants in the *Crumbs homolog 1 (CRB1)* gene lead to severe, childhood-onset retinal degeneration leading to blindness in early adulthood. There are no approved therapies, and traditional adeno-associated viral vector-based gene therapy approaches are challenged by the existence of multiple CRB1 isoforms. Here, we describe three *CRB1* variants, including a novel, previously unreported variant that led to retinal degeneration. We offer a CRISPR-Cas-mediated DNA base editing strategy as a potential future therapeutic approach. This study is a retrospective case series. Clinical and genetic assessments were performed, including deep phenotyping by retinal imaging. In silico analyses were used to predict the pathogenicity of the novel variant and to determine whether the variants are amenable to DNA base editing strategies. Case 1 was a 24-year-old male with cone–rod dystrophy and retinal thickening typical of *CRB1* retinopathy. He had a relatively preserved central outer retinal structure and a best corrected visual acuity (BCVA) of 60 ETDRS letters in both eyes. Genetic testing revealed compound heterozygous variants in exon 9: c.2843G>A, p.(Cys948Tyr) and a novel variant, c.2833G>A, p.(Gly945Arg), which was predicted to likely be pathogenic by an in silico analysis. Cases 2 and 3 were two brothers, aged 20 and 24, who presented with severe cone–rod dystrophy and a significant disruption of the outer nuclear layers. The BCVA was reduced to hand movements in both eyes in Case 2 and to 42 ETDRS letters in both eyes in Case 3. Case 2 was also affected with marked cystoid macular lesions, which are common in CRB1 retinopathy, but responded well to treatment with oral acetazolamide. Genetic testing revealed two c.2234C>T, p.(Thr745Met) variants in both brothers. As G-to-A and C-to-T variants, all three variants are amenable to adenine base editors (ABEs) targeting the forward strand in the Case 1 variants and the reverse strand in Cases 2 and 3. Available PAM sites were detected for KKH-nSaCas9-ABE8e for the c.2843G>A variant, nSaCas9-ABE8e and KKH-nSaCas9-ABE8e for the c.2833G>A variant, and nSpCas9-ABE8e for the c.2234C>T variant. In this case series, we report three pathogenic *CRB1* variants, including a novel c.2833G>A variant associated with early-onset cone–rod dystrophy. We highlight the severity and rapid progression of the disease and offer ABEs as a potential future therapeutic approach for this devastating blinding condition.

## 1. Introduction

**Figure 1:**
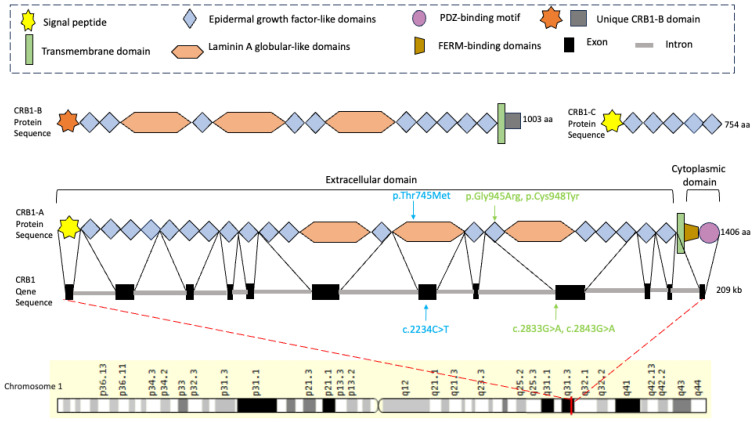
A schematic representation of the *Crumbs homolog 1 (CRB)1* gene and protein structure. The *CRB1* gene is composed of 12 exons which can alternatively be spliced into three isoforms: CRB1-A, CRB1-B, and CRB1-C. The CRB1-A isoform includes nineteen epidermal growth factor-like domains, three laminin A globular-like domains, a signal peptide, a transmembrane domain, and small cytoplasmic domains with FERM-binding domains and a PDZ-binding motif. The CRB1-B isoform has transmembrane domains, extracellular domains (nineteen epidermal growth factor-like domains and three laminin A globular-like domains), and a unique C- and N-terminus. CRB1-C contains the initial six epidermal growth factor-like domains. The green arrows indicate the Case 1 heterozygous variants of c.2843G>A, p.(Cys948Tyr) and a novel missense variant, c.2833G>A, p.(Gly945Arg), in exon 9 of *CRB1.* The blue arrows indicate the mutations in Cases 2 and 3. This c.2234C>T and p.(Thr745Met) are homozygous variants in the CRB1 gene located in exon 7. This figure was created using BioRender (www.biorender.com, last accessed on 7 January 2024).

The Crumbs protein is a large transmembrane protein originally discovered at the apical membrane of Drosophila epithelial cells [1]. It was later found that mutations in Crumbs homolog-1 (CRB1), a human homolog of the Drosophila protein Crumbs, caused retinal dystrophies in humans [2]. The human *CRB1* gene (Cytogenetic location: 1q31.3, OMIM #604210, 209 kb) comprises 12 alternatively spliced exons resulting in three different isoforms of CRB1: CRB1-A, CRB1-B, and CRB1-C. The CRB1-A isoform (1406 aa) contains extracellular domains (nineteen epidermal growth factor-like domains, three laminin A globular-like domains, and a signal peptide), a transmembrane domain, and small cytoplasmic domains (FERM-binding domains and a PDZ-binding motif). The CRB1-B isoform (1003 aa) possesses a transmembrane domain, a large part of the extracellular domain, and a unique C- and N-terminus (Figure 1) [3,4]. The CRB1-C isoform (754 aa) contains the initial epidermal growth factor-like domains of CRB1-A (Figure 1). In the murine retina, CRB1 isoforms represent cell-type-specific expression patterns; CRB1-B localizes exclusively to photoreceptors, while CRB1-A is found in Müller glial cells [5]. There is no evidence of cell-type-specific expression patterns for the CRB1-C isoform in the literature.

**Figure 2:**
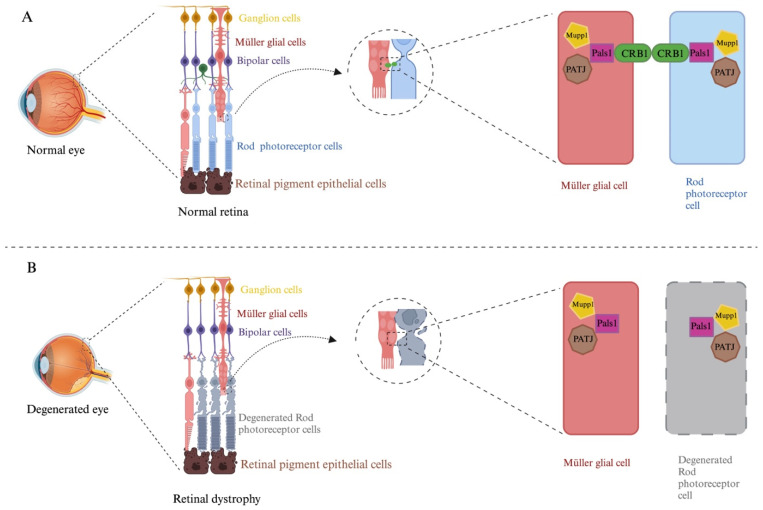
Model of CRB1 localization in human retinal cells. (**A**) The general structure of the retina comprises several cell types: Müller glial cells, bipolar cells, horizontal cells, amacrine cells, retinal ganglion cells, rods, and cones. CRB1 is localized at the subapical region above the adherens junction between the photoreceptor and Müller glial cells as part of protein complexes formed by Lin seven 1 (Pals1), Pals1-associated TJ protein (Patj) and multiple-PDZ domain protein 1 (Mupp1). The CRB1 protein complex plays a crucial role in photoreceptor morphogenesis, apicobasal polarization and adherent junctions in retinal cells. (**B**) A loss of the CRB1 protein leads to retinal dystrophy due to photoreceptor and Müller glial cell degeneration. The figure was created using BioRender (www.biorender.com, last accessed on 7 January 2024).

The *CRB1* gene is expressed in the inner segments of photoreceptors and Müller glial cells in humans. Photoreceptor cells are connected to Müller glial cells via adherens junctions. In adherens junctions, the CRB1 protein is found in complex with proteins associated with Lin seven 1 (Pals1). Pals1 functions as an adaptor protein that binds to the cytoplasmic domain of CRB1 and Pals1-associated TJ protein (Patj) and multiple PDZ domain protein 1 (Mupp1) (Figure 1) [6]. Therefore, this CRB1 protein complex plays a crucial role in photoreceptor morphogenesis, apicobasal polarization, and adherent junctions in retinal cells (Figure 2) [7,8].

Over 150 disease-associated variants leading to a variety of retinal degenerations, most commonly to Leber congenital amaurosis and early-onset rod–cone dystrophy (52%) but also to retinitis pigmentosa (25%), have been described in the *CRB1* gene by the Human Gene Mutation Database (http://www.hgmd.org, last accessed on 7 January 2024) [9]. Hypomorphic alleles, often in-frame deletions, have been shown to cause macular degeneration (MD, 23%), a milder phenotype [10,11,12]. The typical morphological features of *CRB1*-associated inherited retinal degenerations (IRDs) can include an abnormally laminated and thickened retina [13], peripheral exudative retinal telangiectasia (Coats-like vasculopathy) [14], preservation of the para-arteriolar retinal pigment epithelium [15], foveal retinoschisis [16,17], hyperopia [18], nanophthalmos [19], nummular pigmentation [20], and optic disc drusen [21]. Various environmental and genetic factors contribute to the phenotypic characteristics of *CRB1* patients [20,22]. Because of these overlaps and variability in phenotypes, a genotype–phenotype correlation has not been definitively established for *CRB1* patients. Nevertheless, it has been observed that frameshift changes, nonsense mutations [9], aberrant splicing, and a lack of disulfide bonds due to mutated cysteine are related to increased disease severity [10]. In contrast, missense variants or in-frame deletions are associated with milder disease severity [10]. *CRB1*-associated IRDs do not currently have an approved therapeutic option. However, an adeno-associated viral (AAV)-based gene therapy-mediated retinal transgene has proven safe and effective for autosomal [23] and X-linked pathogenic mutations [24] in humans. Over the last ten years, clinical trials of adeno-associated virus (AAV) gene replacement therapies for the *RPE65 (LCA2)* [25], *CHM*-REP1 [26], *RPGR* [24], *CNGA3* [27], *VEGFR1/FLT-1* [28], *ND4* [29], and *MERTK* [30] genes have shown significant promise to prevent Leber congenital amaurosis type 2, choroideremia, *RPGR*-related retinopathy, achromatopsia, wet age-related macular degeneration, Leber hereditary optic neuropathy, and retinitis pigmentosa, respectively. Due to the multiple isoforms of CRB1, gene replacement therapy may not be an optimal treatment option, and alternative approaches are therefore needed. This finding indicates the need for a therapy independent of the CRB1 isoform, such as genome editing technology based on clustered regularly interspaced short palindromic repeats (CRISPR). Our previous study showed that 62% of pathogenic *CRB1* single nucleotide variants can be corrected using CRISPR-Cas-mediated base editing strategies [31].

Typically, *CRB1* retinal degeneration has a rod–cone phenotype. However, this case series describes three individuals with cone–rod dystrophy, a rare, early-onset type of *CRB1* IRD with a severe phenotype which was shown to be caused by pathogenic single-nucleotide *CRB1* variants, including a previously unreported c.2833G>A (p.Gly945Arg) missense variant. We provide an in silico analysis to identify potential treatment strategies using base editing.

## 2. Materials and Methods

This study was conducted following an Oxford University Hospital Institutional Review Board-approved protocol, and all procedures were performed according to the tenets of the Helsinki Declaration [32].

- Patient selection and genetics: This retrospective case series study presents three patients with IRD carrying novel disease-causing *CRB1* variants. Based on the genetic database of Oxford Eye Hospital (Oxford, UK), the patients were identified, and their records were subsequently reviewed. Genetic testing of the patients was conducted by taking DNA blood samples and analyzing them based on direct Sanger sequencing and next-generation sequencing-based retinal dystrophy gene panels.
- Clinical assessment and retinal imaging: A complete ophthalmic examination and imaging were performed on each of the three patients. Details of the clinical assessments, including visual acuity and dilated fundal examinations, were collected. The patients underwent retinal imaging, including color and wide-field photography, fundus autofluorescence (55° and 30°), optical coherence tomography (Spectralis HRA2, Heidelberg Engineering, Heidelberg, Germany), and wide-field fundus imaging (Optos, 200Tx confocal scanning laser ophthalmoscopy camera).
- In silico molecular genetic analysis: To identify previously reported variants in *CRB1*, a search of the Leiden Open Variation Database (LOVD) and ClinVar was conducted (https://databases.lovd.nl/shared/variants/CRB1/unique; accessed on 10 October 2022). The pathogenicity of each variant was classified according to the guidelines of the American College of Medical Genetics and Genomics (ACMG) [33]. Several computational tools were utilised to evaluate functional in silico predictions of variant effects, including Polymorphism Phenotyping version 2 (PolyPhen2), Sorting Intolerant from Tolerant (SIFT), and Mutation Taster [34,35,36]. In order to evaluate the preservation of amino acids between species, Geneious (Version 11.0) and Mutation Taster2021 software were used.
- DNA Base Editing Analysis: To assess whether the patient variants were amenable to DNA base editing, an in silico analysis was conducted. First, the variants were analyzed based on variant type as only single-nucleotide transition variants (G>A, A>G, C>T, T>C) and some transversion variants (G>C, C>G) can be edited using current technology [37,38,39]. Then, available PAM sites were screened and appropriate guideRNAs were designed for the most commonly used constructs, nSpCas9-ABE8e, nSaCas9-ABE8e, KKH-nSaCas9-ABE8e [40], and CasMINI-ABE8e [41], using Benchling software 2022 (San Francisco, CA, USA), GRCh37 (h19) as the reference genome annotation, and ENST00000367400 as the reference transcript. Finally, each designed guide RNA sequence was analyzed for possible unwanted bystander edits and their likely consequences on the amino acid level [42].

## 3. Results

### 3.1. Clinical

**Figure 3:**
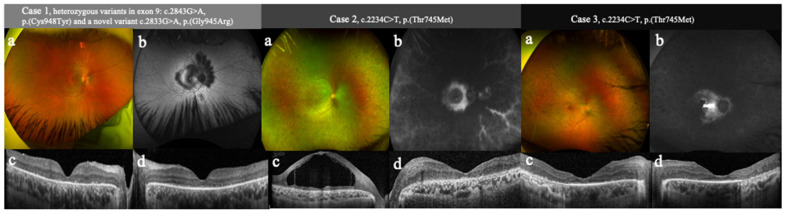
Optos (**a**), wide-field auto-fluorescence (AF) (**b**), and optical coherence tomography (OCT) (right eye (**c**) and left eye (**d**)) images from the three presented cases. Case 1: The optos image shows an unremarkable retinal phenotype (**a**) with an abnormal central hypoautofluorescent pattern seen on AF imaging (**b**) suggestive of a cone–rod dystrophy pattern with predominant macular involvement. The OCT images show abnormal retinal lamination, increased retinal thickness, especially of the retinal nerve fiber layer, and a relatively preserved central outer retinal structure (**c**,**d**). Case 2: The optos image shows non-specific mild–moderate pan-retinal pigmentary retinopathy which is more marked in the periphery (**a**). Fundus autofluorescence shows abnormal hypoautofluorescence with a degree of macular sparing. There is an abnormal autofluorescent ring-like pattern at the macula and hyper-autofluorescent streaks following telangiectic retinal vessels in the periphery representing the preserved perivascular retinal pigment epithelium. The dark center is due to masking by the cystoid spaces combined with RPE atrophy, so the surrounding “bright” AF is likely representing relatively iso-autofluorescent preserved tissue (**b**). The OCT images show a loss of retinal lamination (**c**,**d**), marked cystoid macular edema (**c**), and a significant disruption of the outer nuclear layers and photoreceptor ellipsoid zone (and multiple other laminae) (**c**,**d**). Case 3: The optos image shows even more advanced retinopathy compared to Case 2, with marked pan-retinal pigmentary retinopathy (**a**). Fundus autofluorescence is severely affected by predominant pan-retinal hypoautofluorescence and abnormal macular hyperautofluorescence (**b**). OCT images show abnormal retinal lamination with a reduced distinction between the retinal layers, increased para-foveal retinal thickening (predominantly of the nerve fiber layer), and significant disruption and thinning of the outer nuclear layers, particularly of the foveal photoreceptors and the ellipsoid zone (**c**,**d**).

Case 1 was a 24-year-old male who presented with reading difficulties since childhood and severe photosensitivity but no problems with night vision. Retinal imaging by wide-field optos and fundus autofluorescence (Figure 3a,b) was suggestive of predominant cone involvement with a cone–rod dystrophy phenotype without peripheral pigmentary retinopathy. Optical coherence tomography (OCT) (Figure 3c,d) shows abnormal retinal lamination and increased retinal thickness typical of *CRB1* retinopathy due to the loss of “inter-cellular adhesion” that CRB1 provides to the retinal organization. In addition to being congenital, this poor OCT lamination is thought to have a degenerative component as well [9]. Central outer retinal structures are, in this case, anatomically relatively preserved. The best corrected visual acuity (BCVA) was 60 ETDRS letters in both eyes. Cases 2 and 3 were two brothers, aged 20 and 24, who presented with an early-onset, severe loss of central vision and photophobia. In Case 2, retinal imaging by wide-field optos shows non-specific mild-to-moderate pan-retinal pigmentary retinopathy which was more evident in retinal periphery (a). There is an abnormal autofluorescent ring-like pattern at the macula and hyper-autofluorescent streaks following telangiectic retinal vessels in the periphery (b). In Case 3, an optos image shows even more advanced retinopathy compared to Case 2, with marked pan-retinal pigmentary retinopathy (a). Retinal autofluorescence is severely affected by predominant pan-retinal hypoautofluorescence and abnormal macular hyperautofluorescence (b). At presentation, there was already a loss of retinal lamination (more severe in Case 2) and a significant disruption of the outer nuclear layers, as seen on OCT (Figure 3c,d). Case 2 was also affected with marked cystoid macular lesions (Figure 3c), a common feature of *CRB1* retinopathy, and responded well to treatment with oral acetazolamide. BCVA was reduced to hand movements in both eyes in Case 2 and 42 ETDRS letters in both eyes in Case 3.

### 3.2. Genetic Results and ACMG Classification

Genetic testing for Case 1 revealed compound heterozygous variants. The missense variant c.2843G>A, p.(Cys948Tyr) corresponds to the third Laminin A-like domain of the CRB1 extracellular domain. The c.2843G>A (p.Cys948Tyr) missense variant is one of the most commonly observed variants in the *CRB1* gene in European cohorts [9]. This variant replaces neutral and polar Cysteine with neutral and slightly polar Tyrosine at codon 948 of the CRB1 protein (p.Cys948Tyr). According to the multiple sequence alignment of PolyPhen2, Tyrosine has not been observed at the Cysteine amino acid position in any species, indicating a low tolerance toward this amino acid. The p.Cys948Tyr variant frequency has been reported to be approximately 2:10,000 in the Genome Aggregation Database (https://gnomad.broadinstitute.org/variant/1-197403836-G-A, last accessed on 7 January 2024). Computational tools such as SIFT, Mutation Taster, and PolyPhen2 predict this variant to be deleterious, damaging, and probably damaging, with a score of 0.999 (sensitivity: 0.14; specificity: 0.99). This variant has been classified as pathogenic based on the following ACMG criteria: PS1, PM1, PM2, PM3, PP3, and PP4 (Table 1).

A second variant in Case 1 is a novel c.2833G>A (p.Gly945Arg) missense variant in exon 6 of *CRB1*, corresponding to the third Laminin A-like domain of the CRB1 extracellular domain. This variant replaces Glycine, which is neutral and non-polar, with Arginine, which is basic and polar, at codon 945 of the CRB1 protein (p.Gly945Arg). The high degree of Arginine conservation among a broad range of species suggests limited tolerance for its substitution of Glycine. This variant is not present in population databases (gnomAD: no frequency). Three computational prediction tools, SIFT, Mutation Taster, and PolyPhen2, predicted that c.2833G>A is deleterious, damaging, and probably damaging, with a score of 0.999 (sensitivity: 0.14; specificity: 0.99). This missense change was observed in individuals with inherited retinal disease in a previous study [43]. It has also been reported three times in the ClinVar database, with differing interpretations. Two independent laboratory reports classify the missense change as uncertain significance, while one laboratory report classifies it as likely pathogenic. Due to the conflicting classification of the variant, these evidence criteria cannot be utilized to support pathogenicity. This missense variant has been classified as likely pathogenic due to the application of the following ACMG criteria: PM1, PP3, PP4, PM2, and PM3 (Table 1).

Cases 2 and 3 appeared homozygous for missense variants, c.2234C>T and p.(Thr745Met), in exon 6 of the *CRB1* gene. These variants correspond to the 13th epidermal growth factor-like domains of the CRB1 extracellular domain. This sequence change replaces Threonine, a neutral and polar amino acid, with Methionine, a neutral and nonpolar amino acid, at codon 745 of CRB1 protein (p.Thr745Met). The high evolutionary conservation of Methionine across a broad range of species suggests a low tolerance for this amino acid to change to Threonine. The p.Thr745Met variant frequency has been reported to be approximately 3:10,000 in the Genome Aggregation Database (http://www.gnomad-sg.org/variant/1-197398749-G-A?dataset=gnomad_r2_1, last accessed on 7 January 2024). When the c.2234C>T, p.(Thr745Met) variant was analyzed using three computational predictive tools (SIFT, Mutation Taster, and PolyPhen2), it was predicted to be deleterious, damaging, and probably damaging, with a score of 1 (Sensitivity = 0, Specificity = 1). The variant has been classified as pathogenic based on the application of the following ACMG criteria: PS1, PM1, PM2, PM3, PP1, PP2, PP3, and PP4 (Table 1).

### 3.3. In Silico Analysis of CRISPR-Cas-Mediated Genome Editing Strategies

Case 1 was found to be compound heterozygous for two missense variants: c.2843G>A, p.(Cys948Tyr) and c.2833G>A, p.(Gly945Arg). As G-to-A transition variants, both could, in theory, be corrected with an ABE introducing the A-to-G edit and thus reverting the variants back to the wild type. For the c.2843G>A, p.(Cys948Tyr) variant, the in silico analysis revealed no suitable PAM sites for the nSpCas9-ABE8e, nSaCas9-ABE8e and CasMINI-ABE8e constructs, but a PAM site was found for the KKH-nSaCas9-ABE8e construct. A guideRNA for this base editor was then determined: TTAG[G>A]TATTGCAAATGCTGTTtttaat (the guideRNA sequence is marked in uppercase letters, the variant is indicated in brackets, the editing window is underlined, and the PAM site is indicated with lowercase letters) (Table 2). Unfortunately, the safety of this strategy is likely limited by the four predicted bystander edits c.2843-2A>G, p.Ile949Val, p.Ala950Ala, and p.Asn951Asp, of which one is very likely a splicing variant and two amino are acid-changing missense variants.

The in silico analysis of the second variant observed in Case 1, c.2833G>A, p.(Gly945Arg), revealed no available PAM sites for the nSpCas9-ABE8e and CasMINI-ABE8e constructs, while one was observed for the nSaCas9-ABE8e and KKH-nSaCas9-ABE8e. Again, a guide RNA was designed accordingly: CTTCAA[G>A]GATTTGAATGTAGGtagagt, revealing two likely bystander edits, p.Q944Q/R and p.G945G/R, that would again reduce the safety of this approach.

Cases 2 and 3 were found to be homozygous for the c.2234 C>T, p.(Thr745Met) variant that could be corrected with an ABE in the reverse strand. A PAM site analysis indicated no available PAM sites for the CasMINI-ABE8e, nSaCas9-ABE8e, and KKH-nSaCas9-ABE8e constructs, while one was observed for the nSpCas9-ABE8e, and an appropriate guide RNA was again designed: TTGAAGC[G>A]TTCGGACAAACAtgg (Table 2). This approach likely introduces a bystander edit as well (p.L746P/L).

## 4. Discussion

Depending on the level of residual CRB1 activity and the genetic background, *CRB1* mutations can cause severe congenital and early-onset retinal dystrophies, including Leber congenital amaurosis, retinitis pigmentosa, and cone–rod dystrophies. Here, we describe three cases of *CRB1*-associated retinal degeneration, all presenting with cone–rod dystrophy in early childhood. Case 1 was a 24-year-old male with a clinical phenotype typical of *CRB1* retinopathy. Genetic testing showed two variants in exon 9: c.2843 G>A p.Cys948Tyr and a novel variant, c.2833G>A p.Gly945Arg. An in silico prediction confirmed that the novel variant is classified as likely pathogenic according to the ACMG criteria. Cases 2 and 3 were two brothers, ages 20 and 24, who showed severe disruption of the central outer nuclear layers at the time of presentation. Genetic testing confirmed two c.2234 C>T variants in exon 7 in both brothers. The most frequently reported *CRB1* mutations in both the LOVD and ClinVar databases, located in exon 7 and exon 9, correspond to the length of these exons relative to the entire coding sequence [31]. All of our cases represent phenotypes of a more severe spectrum due to the two missense variants. A previous study demonstrated that individuals with one missense variant exhibit a milder phenotype, while those with two missense variants experience a more severe phenotype due to the higher impact on the protein function or structure of isoforms CRB1-A and CRB1-B [10].

The use of recombinant adeno-associated viral vectors in gene therapy has been safe and resulted in long-lasting expression in numerous studies. Initial challenges arose due to the *CRB1* transcript being very close to the maximum packaging size of an rAAV and also the presence of multiple CRB1 isoforms. On the other hand, proof-of-principle studies for treating *CRB1*-associated IRDs showed efficient *CRB1* expression but adverse effects on retinal activity in *CRB1* murine models, which could be caused by the replacement of the CRB1-A isoform but not the other CRB1-B isoform [44,45,46]. Nonetheless, a clinical trial with AAV5.CMV.h*CRB2* recently started, and results are awaited in anticipation. There is, however, an urgent need for a therapy independent of the CRB1 isoform, such as genome editing technology based on clustered regularly interspaced short palindromic repeats (CRISPR). Early generations of CRISPR technology relied on the RNA-guided CRISPR-Cas nuclease system. By recognizing and binding host DNA and a Cas-RNA complex, double-stranded breaks can be induced by the catalytic domains of the Cas endonuclease that are then repaired by non-homologous end joining or homology-directed repair. The non-homologous end joining causes frameshift mutations, premature stop codons, and gene knockouts within the target zone by arbitrary insertions and deletions. While homology-directed repair produces precise changes to DNA utilizing an exogenous template, the procedure is restricted to active dividing cells, unlike post-mitotic cells such as photoreceptors and Müller glial cells [47,48]. Base editing was developed to overcome the limitations of the RNA-guided CRISPR-Cas nuclease system. Among the base editing methods, the cytosine base editor (CBE) causes transitions from C-G to T-A, and the adenine base editor (ABE) causes transitions from A-T to G-C [49]. The limitation of base editing to perform transversion was addressed by glycosylase base editors to perform C-to-G transversions [50]. The base editors have recently been optimized to improve target scope using SpCas9 variants and homologues with alternative PAM requirements, such as ScCas9 [51], as well as relaxed PAM requirements, such as KKH-SaCas9 [52] and CjCas9 [53]. However, base editing still has important limitations, including the inability to correct insertions, deletions, and many types of transversion point mutations—all of which could, however, be corrected with another type of CRISPR-Cas9-mediated gene editing termed prime editing [54]. Recent studies have demonstrated the potential of CRISPR activation to treat various loss-of-function diseases [55]. CRISPR activation of the *CRB1* gene might boost the expression of a wild-type gene or another isoform to restore proteins close to their functional level in haploinsufficient cases (mutant or absent in one diploid copy of the gene). Increasing the endogenous activation of *CRB2* as an equivalent homolog of *CRB1* by CRISPR activation can be an efficient treatment in recessive cases (mutant or absent in two diploid copy of the gene). According to our previous report, 62% of *CRB1*-associated retinal degeneration variants can be corrected by a base editor. Therefore, this approach offers a promising treatment strategy for *CRB1*-related retinal degeneration [31]. In this report, DNA base editing could, in theory, be used to correct the c.2843G>A CRB-1 variant using the KKH-nSaCas9-ABE8e base editor and the c.2833G>A variant using the nSaCas9-ABE8e, KKH-nSaCas9-ABE8e base editor in Case 1 (Table 2). In Cases 2 and 3, DNA base editing could theoretically correct the c.2234C>T CRB-1 variant using the nSpCas9-ABE8e base editor (Table 2). All three variants could, in theory, be corrected, and available PAM sites were found for all of them and the respective guide RNAs were designed. However, the safety and thus feasibility of this therapeutic approach would likely be limited by the predicted unwanted bystander edits that could thwart the treatment effect completely. Similar challenges have been reported in base editing, with over half of the guide RNAs for different constructs introducing potentially harmful bystander edits [42]. These bystander effects can influence cellular function by introducing unexpected changes in gene expression, protein function, or regulatory networks, potentially impacting cell viability and proliferation or even leading to unintended consequences such as cancerous transformations. The bystander effect in base editing could potentially impact the progression of visual disorders via unintended changes in nearby genes or regulatory elements, potentially affecting the efficacy and safety of the therapy. Retinal cells could develop unintended alterations in gene function or expression, affecting visual processing or integrity. On the other hand, bystander effects may increase the risk of tumorigenesis in the retina or adjacent tissues if unintended genomic alterations occur in genes associated with cell proliferation or tumor suppression. This could potentially worsen the progression of visual disorders or lead to additional complications [56]. Overall, minimizing bystander effects and ensuring the specificity and safety of base editing techniques are critical considerations for the development of effective therapies for visual disorders. Developing more precise base editors with enhanced target specificity, optimizing efficient and localized delivery methods, and designing gRNAs with high specificity for the target site are some ways to minimize bystander effects and improve the safety and efficacy of base editing technologies for various applications [57,58].

This proof-of-concept evaluation of base editing shows the potential promise for the clinical application of gene editing for *CRB1* mutations. The science must move forward from the in silico analysis of potential *CRB1* base editing therapeutics to in vitro studies on *CRB1* patient iPSC-derived retinal organoids [59] and in vivo studies on naturally occurring or engineered mouse models [60].

In order to design an optimal base editor construct, a strategy for delivery to the cell should also be considered. AAVs are a leading platform for gene delivery in the treatment of retinal dystrophy. They are less toxic, can transduce both dividing and non-dividing cells, do not integrate into the host genome, and display distinct cell tropisms. Various strategies have been developed to address AAVs’ limited package capacity, such as utilizing a minimal CMV promoter [61], dual- or triple-AAV approaches [62,63], and increasing packaging capacity [62]. Each type of AAV capsid results in a distinct expression pattern in retinal cells. Of the distinct AAV capsids, AAV2.5 with the minimal CMV promoter (AAV5.CMVmin.h*CRB1*) proved to be successful in transducing *CRB1* patient-derived retinal organoids. Therefore, it could be a potential option for the genome editing gene therapy of retinal dystrophies related to *CRB1* [64]. In addition, non-viral mediated approaches like nanoparticles have an unlimited gene packaging capacity, which is advantageous for large genes such as CRB1. Nanoparticles can drive gene expression in mice with a similar scale and duration as AAVs [65]. Nanoparticles have been identified as a safe drug-delivery system for the eyes in mice and primates. However, no successful human clinical trials have been conducted using nanoparticles [66].

In conclusion, the promise of base editing in severe progressive retinal degeneration with congenital retinal abnormalities such as *CRB1* lies in its potential to address the underlying genetic mutations responsible for these conditions [67,68,69]. Base editing offers a precise and targeted approach to correcting specific nucleotide changes associated with these disorders, potentially halting or even reversing disease progression at the genetic level. This holds significant promise for patients who currently have limited treatment options and face the prospect of progressive vision loss or congenital visual impairments. However, there are several limitations to consider. First, base editing is primarily effective for correcting certain types of mutations, such as single-nucleotide substitutions, which may not cover the full spectrum of genetic abnormalities underlying retinal diseases. Additionally, the efficient delivery of base editing components to the retina remains a challenge, particularly for in vivo applications win which targeted delivery to specific retinal cell types is necessary. Furthermore, the long-term safety and efficacy of base editing in the complex and delicate environment of the retina require thorough evaluation to ensure minimal bystander effects and potential risks. Ongoing research and advancements in base editing technology offer hope for addressing these challenges and unlocking the therapeutic potential of genome editing in treating serious progressive retinal diseases with congenital retinal abnormalities [70,71]. With further refinement and optimization, base editing could provide personalized and precise medicine approaches tailored to individual patients’ unique genetic profiles, ultimately improving their quality of life and visual outcomes. Despite some limitations, the potential benefits of base editing are significant and could drastically improve the treatment of these diseases.

## Figures and Tables

**Table 1:** The characteristics and classification of the *Crumbs homolog 1 (CRB1)* variants.

Case Number	Variant	Protein Consequence	Frequency	ACMG Classification and Criteria	Pathogenicity
Case 1	c.2843G>A,	Cys948Tyr	2:10,000	PS1, PM1, PP3, PP4, PM2, and PM3	Pathogenic
	c.2833G>A,	Gly945Arg	No frequency reported	PM1, PP3, PP4, PM2, and PM3	Likely Pathogenic
Case 2 and Case 3	c.2234C>T Homozygous	Thr745Met	3:10,000	PS1, PM1, PM2, PM3, PP1, PP2, PP3, and PP4	Pathogenic

**Table 2:** CRISPR-Cas-mediated base editing approaches for *CRB1*.

Case Number	*CRB1* Variants	Base Editing Construct	Designed gRNA with Target Mutation Site in Red, PAM Site in Green, and Known Editing Window Highlighted in Yellow
1	c.2843G>A c.2833G>A	KKH-nSaCas9-ABE8e nSaCas9-ABE8e; KKH-nSaCas9-ABE8e	TTAG[G>A]TATTGCAAATGCTGTTTTTAAT CTTCAA[G>A]GATTTGAATGTAGGTAGAGT
2 and 3	c.2234C>T	nSpCas9-ABE8e	TTGAAGC[C>T]TTCGGACAAACATGG

## Data Availability

All data generated or analyzed during this study are included in this published article.
